# China’s new policy for healthcare cost-control based on global budget: a survey of 110 clinicians in hospitals

**DOI:** 10.1186/s12913-019-3921-8

**Published:** 2019-02-01

**Authors:** Jianzhou Yan, Hui-Heng Lin, Dan Zhao, Yuanjia Hu, Rong Shao

**Affiliations:** 1 School of International Pharmaceutical Business, China Pharmaceutical University, Nanjing, Jiangsu People’s Republic of China; 2 The Research Center of National Drug Policy and Ecosystem, China Pharmaceutical University, Nanjing, Jiangsu 211198 People’s Republic of China; 3 State Laboratory of Quality Research in Chinese Medicine, Institute of Chinese Medical Sciences, University of Macau, Avenida da Universidade, Room 2053, N22, Taipa, 999078 Macau People’s Republic of China

**Keywords:** China healthcare budget crisis, Healthcare cost-control policy, Hospitals’ healthcare cost-control actions, Clinician survey, Healthcare performance, Patient satisfaction

## Abstract

**Background:**

The increasing cost on healthcare exposes China’s healthcare budgets and system to financial crisis. To control the excessive growth of healthcare expenditure, China’s healthcare reforms emphasize the control of the global budget for healthcare, which leads to the release of relevant policy and a series of cost-control actions implemented by different hospitals. This work aims to identify the effects brought by the cost-control policy and actions via surveying and analysing feedback from clinicians.

**Methods:**

Questionnaires on the cost-control policy and actions were designed for surveying 110 clinicians in hospitals from different regions of China. The data on the implementation of the cost-control actions and doctors’ feedback on these actions were analysed using descriptive statistics. Pearson’s chi-squared tests were performed to detect associations between doctors’ opinions and specific cost-control actions. A value of *p* < 0.05 was considered statistically significant. Association relationships between doctors’ opinions and cost-control actions were modelled into network models, and key factors were identified in a multi-variate framework. Last, we visualized our resultant data using a network model, and further multi-variate analysis was performed.

**Results:**

There were three main findings. (1) The cost-control policy has been widely implemented in the sampled hospitals in different regions of China, with more than 80% of those surveyed acknowledging that their hospitals take actions of reducing average prescription fees for outpatients, drug costs, and in-hospitalization durations. (2) Most doctors have a negative view of some cost-control actions; this is mainly due to concerns about the effects of these actions on the doctors’ own healthcare performance and patient satisfaction. (3) Cost-control actions that had a significant impact on doctors’ performance included limiting average prescription fees for outpatients and limiting the use of examinations/drugs/surgeries. Decreased patient satisfaction was associated with fewer admissions of critically ill patients, reduced use of brand-name drugs, and increased total costs to patients due to increased frequencies of visits to the hospitals.

**Conclusions:**

Cost-control actions implemented in hospitals in response to the government’s policy to reduce its national healthcare budget affect both doctors and patients in several ways. Moreover, the cost-control policy and actions can be improved.

**Electronic supplementary material:**

The online version of this article (10.1186/s12913-019-3921-8) contains supplementary material, which is available to authorized users.

## Background

In China, the majority of the medical insurance for citizens is the basic medical insurance scheme, and the alternative commercial medical insurances are available as the minority. According to China’s medical insurance framework and system established in 2007, basically, the citizens are now able to reimburse a large part of the healthcare costs from diseases’ treatments and pharmacotherapies. In China, the payments of healthcare costs are associated with patients’ medical insurance types. Briefly, for patients with the basic medical insurance, the medical insurance will cover or reimburse patients’ costs on the basic healthcare options/treatments, i.e., this part of healthcare expenditure will be paid to hospitals by the management institutions of healthcare insurance funds. These institutions could either be insurance companies or governmental agencies. The partial revenues of hospitals from these institutions will transfer to the salary or bonus of the clinicians/physicians in hospitals. For costs not covered by the basic medical insurance, patients have to pay the bill from their own pocket. While for patients with other kinds of medical insurance, such as the advanced commercial plans or the corporate plans of the medical insurance, the amount of bill that has to be paid by patients themselves varies according to the coverage levels of the healthcare options in their own medical insurance.

While as is the case in many other countries, China’s healthcare budget and system are now facing financial crisis due to increasing healthcare costs. Currently, in China, the basic medical insurance scheme is benefiting more than 1.3 billion (95% of the country’s total population) of its citizens by partially covering citizens’ healthcare fees using the country’s healthcare funds [1]. However, China is rapidly stepping into the aging society. In 2015, it was reported that 14% of China’s aging population required familial support; this figure was double the global standard of 7% [2]. Therefore, the demand for healthcare quickly increases [3–5], so do healthcare expenditure and reimbursement charges of patients’ medical insurances from funds. Because the increase of healthcare expenditure is much faster than that of the incomes of healthcare funds and budgets, the healthcare system is experiencing a financial crisis.

To control the excessive growth in healthcare expenditure, the government of China tried to adopt the global budget program and developed a cost-control policy in 2012. Since then on, the healthcare institutions, mainly hospitals in China started the trials and taking actions on cost-control of healthcare activities. The policy includes a series of cost-control actions or policies for hospitals. Briefly, the government aims to control or reduce the reimbursement quota and amount of citizens’ healthcare costs covered by medical insurances, as well as reduce the costs on healthcare activities, so as to ease the financial stress. The policy sets upper limits both for healthcare budget funds and the total reimbursement fee covered by medical insurances; in other words, if extra options or activities of healthcare beyond the scope of the cost-control policy and actions happened, the insurance firms or the healthcare funds’ management institutions will not pay these extra fees to hospitals. Namely, if extra healthcare fees are charged, the hospitals have to either cover these charges or send the bills to patients. In such way, the policy and the budget control system force the hospitals to control healthcare costs [6, 7].

Global budget has been used by several countries, such as Canada and Australia to control healthcare costs. So far, there exist numerous studies about the global budget. Most of them focused on the global budget’s system itself, e.g., the payment, the paying methods, and the measurements [8], or the outcomes of the global budget implementation, e.g., the changes on healthcare costs, and the quality and efficiency of the healthcare [9, 10]. For instance, Du et al. considered that, there were multiple ways for estimating the global budget and the appropriate one should be chosen based on the local laws and regulations of the involved local agencies [11]. Cheng et al. analysed the responses of hospitals to the global budget and found that hospitals attempted to increase per-case expense claims to protect the reimbursements from possible discounts under a global budget [12]. Most of existing studies are either theoretic works or case analyses based on single case [13].

With a population of over 1.3 billion, China is the world’s largest developing country, and its healthcare cost-control policy and actions for addressing the budget crisis have attracted global attention. The policy and actions have large impacts, both on the healthcare of about one-fifth of the world’s population and the sustainable growth of the healthcare market/industry in China, and hence they are widely debated in the society. Several reports claim that similar cost-control policies and methods have effectively controlled healthcare costs in other countries [14–16]. Other studies also report the positive results and viewpoints on the policy after its implementation in hospitals in China. E.g., these results include preventing the over-use of drugs such as antibiotics, which are causing drug-resistance, and inhibiting the behaviours of excessive medical treatments [17]. However, the opposite and negative opinions exist as well. In some reports, the policy received criticism. There are reports questioning the cost-control effects of the policy as well as its effects on healthcare quality. It is said that the changes of the payment system in the policy give less incentives to healthcare staff so there working motivation are weakened. Hence, the policy reduces the quality of healthcare services, such as, accepting more non-local patients (who do not fall in the scope of the cost-control policy and actions) but less local patients with medical insurance coverage, rejecting critically ill patients, and persuading patients to pay for costly medications, examinations, and therapies [18–21].

Despite the debate surrounding the policy, most studies in existing literature are either theoretic works or case analyses based on single case [8–13]. Especially, there is a lack of first-hand data and analysis from a real-world survey of clinicians in hospitals who are not only the first-line implementers of the cost-control policy but also the bridge that connects healthcare fee payers and providers. To address this gap, we surveyed 110 clinicians in different hospitals to determine the level of implementation of the cost-control actions in hospitals, detect doctors’ opinions to these actions, and identify the factors that influence doctors’ opinions regarding these actions.

As aforementioned, although many studies have analysed or evaluated the policy in terms of its efficacy for cost-control or its effects on health economics, few studies have considered doctors’ perspectives regarding the policy. Our work is a study based on the first-hand data and the real-world survey of the clinicians. Compared with other studies, one feature in this study is the focus on the clinicians’ opinions and attitudes, which are essential in the global budget cost-control policy and actions in terms of clinicians’ role as the direct implementers of the global budget cost-control. In addition, our study emphasizes on healthcare quality affected by the policy instead of other aspects like the financial matters or economic values. Doctors directly perform the medical or healthcare practices and hence are the ones directly carrying out the cost-control policy and actions to patients. While conducting their practice, doctors can clearly recognize what changes are brought by the cost-control policy. Moreover, doctors are the closest to patients among medical staff and thus understand patients’ responses to cost-control actions. Thus, doctors’ feedback and opinions are valuable indicators for studying and evaluating the cost-control policy and identifying its positive and negative outcomes. These reasons are why doctors were chosen for the survey in this study. Through our survey and analysis, we can examine the implementation of the cost-control policy and actions and identify the defects of the current policy and actions to develop improvements to and revisions of the policy, which can significantly affect nation-wide healthcare reform.

## Methods

### Questionnaire and doctor survey

Questionnaires were designed to identify doctors’ opinions towards the cost-control policy and actions and the effects of the policy and actions on the healthcare performance and quality, as well as the rights of doctors and patients (Additional file 1). The questionnaire was developed according to prior studies reviewed and the research purposes of this work. In more details, we would like to address the following key questions. First, how well was the global budget/cost-control policy implemented? Second, how did hospitals respond to the cost-control policy and what detailed actions in doctors’ healthcare practice are designed for cost-control purpose. Third, what are the effects on healthcare quality brought by the cost-control actions. With these core question points in mind and we designed the questionnaire. Note that questions for asking doctors about the thoughts and feelings of the patients were also developed, though such questions would be the indirect survey on patients’ thoughts towards the cost-control policy and action. While given the continuous debate surrounding the policy, and the lack of first-hand data and real-world survey on the issue, it is still of great significance to have a first look at patients’ responses on the policy from physicians’ perspective. Also, instead of the Likert scale responses, we showed the dichotomous responses (Yes/No or Agree/Disagree) for most of the questions because of the following reasons. Initially, the dichotomous answers were necessary and clear enough for this pilot survey as an initial work to fill in the gap on the understanding of the effects of the cost-control policy and actions. Moreover, 110 physicians were interviewed, and because of the limited size of interviewed physicians, it is difficult to generate high-resolution significant results, especially by bivariate and network-based analysis. Thus, the dichotomous responses were used in this article. Last, we tested the validity and reliability of our questionnaire. To carry out the content validity test, we firstly consulted external experienced consultants about questionnaire design. These consultants remarked that our questionnaire widely covers various issues of the cost control policy and actions and meets the research purpose well with sufficient validity.

Moreover, we invited 6 experts with more than 25 years of professional experience, specialized in different professions and domains including public administration, clinical healthcare, medical policy research, drug policy administration, public health administration, and clinical pharmacy to make a quantitative assessment of content validity index (CVI). Two indicators were used to measure the scale-level CVI. The first indicator reflects the universal agreement from the expert raters, which is equal to 0.897. The second indicator calculates the mean value of all item-level CVI. In our case, the value is 0.982. The results above indicate the good content validity of our questionnaire data. On the other hand, we calculated the Kuder-Richardson 20 coefficient for the reliability test. The final coefficient value is 0.7985, indicating a good and reasonable reliability of our questionnaire data.

In the sampling stage, the convenient sampling was employed to this cross-sectional survey research. First, we selected Beijing, Shanghai, Tianjin, Nanjing, and Chengdu as sample regions. These are the earliest cities to implement the global budget scheme as the first-round trial cities listed by the government [4, 5]. Moreover, four to five hospitals at different levels in each city were selected as sample hospitals. Totally, 21 hospitals were involved in this study. In the hospitals selected, physician sample was taken in terms of their availability, professional experience, and willingness. We assessed the qualifications of doctors easy to contact or to reach. The experienced doctors willing to join the one-hour long face-to-face interview were invited to fill in the questionnaire. We distributed the questionnaires to 110 clinical doctors in total. Of the 110 questionnaires, 107 (97%) were valid copies which contained complete answers and information, and three copies were incomplete and hence excluded from the data analysis. Among the 107 valid copies, each city’s hospitals (i.e., hospitals in Beijing, Nanjing, Chengdu, and Tianjin) contributed 20 valid copies. For Shanghai’s hospitals, 27 valid questionnaires were collected. Most of the clinical doctors that were surveyed were senior professionals and hence had extensive experience working in healthcare. Therefore, their opinions clearly reflected the changes brought about by and the effects of the cost-control policy and actions.

### Statistical and network analyses

#### Descriptive analysis

In order to generate an overview of the distribution of the surveyed data, we performed descriptive statistical analysis of our data, which included data regarding the cost-control actions taken by hospitals, as well as doctors’ opinions and preferences regarding these actions.

### Bivariate analysis

Furthermore, upon manual data collection, curation, and integration, Pearson’s chi-squared tests were performed to detect the statistical independency between two events. Here, events refer to the questions from the questionnaires. According to the “Yes/No” or “Agree/Disagree” answers indicated by the doctors, data were sorted and loaded into the SPSS statistics 21 software for the chi-squared test. Three thresholds were set for the results of the statistical significance: the *p*-value < 0.1, 0.05, or 0.001.

### Network construction and multivariate analysis

In order to provide an overview of our results on the system perspective, we employed the network model to visualize our results. In the constructed directed network, the nodes are the events from the chi-squared tests, and the association relations between events were obtained from the statistical results. If a test result was significant, then two events were associated and hence two nodes in the network were linked by an edge. The network model is partially similar to the Bayesian network, which can reflect the potential cause-effect relationships between nodes.

Nodes were coloured differently to identify different types of nodes. The cost-control actions in the network were assigned the blue colour, whereas the doctors’ opinions were the assigned grey colour. Also, bigger sizes were assigned to nodes with more degrees to indicate the important nodes in the network. Last, edges linking different nodes were assigned different weights according to the results of the statistical significance. Hence, a greater statistical significance creates a thicker edge between two nodes. The visualization of the network and centrality analysis were done on Gephi [22].

## Results

### Hospitals’ actions for cost-control

In this work, the cost-control actions were surveyed. The cost-control actions taken by hospitals are diverse, but the most commonly taken action, which was reported by 94 of the 107 surveyed doctors, was limiting average prescription cost in outpatient service and proportional costs of total medical expenses (Table 1). Other frequently reported and top-ranked actions include limiting the duration of hospitalization, average cost in hospitalization, usage of top-ranked drugs, and usage of examination/drug/surgery, as seen in Table 1.

**Table 1:** Frequently taken and top-ranked cost-control actions by hospitals

Class	Cost-control actions from hospital	Frequency	Percentage
High-frequency actions	Limit average prescription cost in outpatient service	94	87%
	Limit the proportional cost of total medical expenses (e.g., the proportion of drug costs)	94	87%
	Limit the duration of hospitalization	93	86%
Moderate frequency actions	Limit average cost in hospitalization	75	70%
	Regularly rank and limit the use of top-ranked drugs	74	69%
	Limit the conditions for the usage of examination/drug/surgery	73	68%
	Limit cost of treating single kind of disease	58	54%
Low frequency actions	Limit costs and amounts of examinations/drugs/surgery prescriptions	44	41%
	Limit the cost of examinations	30	28%

### Doctors’ opinions and feedback on cost-control actions

We surveyed doctors’ opinions and feedback on a series of the cost-control actions implemented by hospitals. As seen in Table 2, most doctors speak negatively regarding the implemented actions. On one hand, these actions affect the healthcare performance of doctors. On the other hand, the actions also lower patient satisfaction by reducing patients’ medical resources and thus increasing the healthcare expenses that patients must pay. The most serious outcome is that these actions worsen the relation between doctors and patients.

**Table 2:** Doctors’ opinions about the cost-control actions

Question category	Questions for doctors	No. of Yes/Agree	Percentage of Yes/Agree
Healthcare performances or quality.	Hospitals’ cost-control actions affect doctors’ healthcare performance.	86	80%
	Hospitals’ cost-control actions seriously limit doctors’ healthcare performance.	83	77%
	Hospitals’ cost-control actions are irrational.	71	66%
Patients’ circumstances (observed by doctors).	Indirect costs of patients for visiting hospitals increase (time, transport fees, etc.).	87	81%
	Less medical resources for patients.	56	52%
	The average cost of healthcare paid by patients increases.	24	22%
	The total cost of healthcare paid by patients increases.	60	56%
Relationship between doctors and patients.	Lower patient satisfaction.	92	85%
	Worsen the relationship between doctors and patients.	93	86%

### Factors relevant to doctors’ opinions and feedback

Using descriptive statistical analysis, we identified doctors’ opinions about the cost-control actions. In order to further identify the reasons for or causes of these opinions, we performed extensive bivariate analysis to identify factors associated with doctors’ feedback. The results are shown in the Tables 3, 4 and 5 below. The columns of Event A refer to the doctors’ feedback, and the columns of Event B are the doctors’ opinions of the cost-control actions implemented by hospitals.

**Table 3:** Statistical significance of Chi-squared tests for independency between doctors’ healthcare performance and hospitals’ cost-control actions

Event A	Event B	*P*-value
Hospitals’ cost-control actions affect doctors’ healthcare performance.	Limit average prescription cost in outpatient service.	9·50 × 10^−10^***
	Limit average cost in hospitalization.	0·0326**
	Limit the cost of examinations.	0·0469**
	Limit costs and amounts of examinations/drugs/surgery prescriptions.	0·0730*
	Hospitals accept fewer critically ill patients.	0·0522*
Hospitals’ cost-control actions seriously limit doctors’ healthcare performance.	Limit the cost of treating single kind of disease.	0·0203**
	Limit costs and amounts of examinations/drugs/surgery prescriptions.	0·0684*
	Limit the conditions for the usage of examinations/drugs/surgery.	0·00747***
Hospitals’ cost-control actions are irrational.	Limit average cost in hospitalization.	0·0585*
	Regularly rank and limit the use of top-ranked drugs.	0·0720*
	Worsen the relationship between doctors and patients.	0·00925***

Note: *, **, and *** stand for significance of 10, 5, and 1%, respectively

**Table 4:** Results of Chi-squared tests for independency between patients’ circumstances and cost-control actions or other facts

Event A	Event B	*P*-value
Less medical resources for patients.	Higher frequency of visiting hospitals by patients.	0·0305**
	Lower patient satisfaction.	4·38 × 10^−6^***
	The total cost of healthcare paid by patients increases.	0·00410***
	Indirect costs of patients for visiting hospitals increase (time, transport fees, etc.).	0·0679*
	Hospitals accept fewer critically ill patients.	2·77 × 10^−6^***
	Hospitals’ cost-control actions increase staff workloads.	0·0812*
	Hospitals’ cost-control actions seriously limit doctors’ healthcare performance.	7·98 × 10^−4^***
	The limits on drug prescriptions affect the doctors’ healthcare performance.	0·00601***
The average cost on healthcare paid by patients increases.	Limit average prescription cost in outpatient service.	0·0189**
	Limit cost of treating single kind of disease.	0·0396**
	Limit costs and amounts of examinations/ drugs/surgery prescriptions.	0·0854*
	The total cost on healthcare paid by patients increases.	3·91 × 10^−8^***
	Indirect costs of patients for visiting hospitals increase (time, transport fees, etc.).	0·0142**
The total cost on healthcare paid by patients increases	Higher frequency of visiting hospitals by patients.	0·0904*
	Hospitals’ cost-control actions seriously limit doctors’ healthcare performance.	0·00722***
Indirect costs of patients for visiting hospitals increase (time, transport fees, etc.).	Limit the conditions for the usage of examinations/drugs/surgery.	0·0134**
	Hospitals accept fewer critically ill patients.	0·0010***
	Shortened duration of prescribed medication.	0·00530***
	The total cost of healthcare paid by patients increases.	0·00150***

Note: *, **, and *** stand for significance of 10, 5, and 1%, respectively

**Table 5:** Results of Chi-squared tests for independency between doctor-patient relationship and relevant cost-control actions

Event A	Event B	*P*-value
Lower patient satisfaction.	Limit the cost of examinations.	0·043**
	Limit the conditions for the usage of examinations/drugs/surgery.	9·23 × 10^−4^***
	Reduce the use of brand-name drugs.	0·0529*
	Increase the doctors’ workloads (e.g., have to explain more about why they made the healthcare decisions to patients).	0·0413**
	The total cost on healthcare paid by patients increases.	3·76 × 10^−4^***
	Hospitals accept fewer critically ill patients.	0·00172***
	Hospitals’ cost-control actions increase staff workloads.	0·0438**
	Hospitals’ cost-control actions seriously limit doctors’ healthcare performance.	0·00503***
Worsen the relationship between doctors and patients.	Limit the duration of hospitalization.	0·0653*
	Limit the proportional cost of total medical expenses (the proportion of drug costs, etc.).	0·00379***
	The total cost on healthcare paid by patients increases.	0·00942***
	Hospitals’ cost-control actions increase staff workloads.	2·19 × 10^−6^***
	Less medical resources for patients.	0·0432**

Note: *, **, and *** stand for significance of 10, 5, and 1%, respectively

First, we performed chi-squared tests to examine the statistical independency between doctors’ healthcare performance (i.e., the doctors’ opinions regarding the question category of “healthcare performance”) and cost-control actions taken by hospitals.

Our analysis indicates that many doctors consider that the cost-control actions implemented by hospitals, such as limiting average prescription cost in outpatient service, limiting average cost in hospitalization, and limiting the conditions for the usage of examinations/drugs/surgery, are irrational and limit their performance in providing healthcare to patients (Tables 1 and 3). According to the doctors’ responses, among all the types of cost-control actions, the act of limiting average prescription cost in outpatient service is one of the actions most frequently taken by hospitals and is strongly correlated with the affected performance of healthcare by doctors (Table 1). While the act of limiting the cost of examinations cuts costs down, limited examination resources affects doctors’ ability to make accurate diagnoses, which affects therapeutic decisions and outcomes. Obviously, this significantly limits doctors’ healthcare decisions and is a great risk to patients.

Further analysis identified that, the cost-control actions are limiting the doctors’ healthcare performance to some extent. Among the diverse cost-control actions, three actions were considered the most restrictive of doctors’ healthcare performance: limiting the conditions for the usage of examinations/drugs/surgery, limiting the costs and amounts of examinations/drugs/surgery prescriptions, and limiting the cost of treating single kind of disease.

Second, we analysed the statistical data for statistical significance. Through this analysis, we identified the association between the resulting circumstances of patients (items in Table 2’s “patients’ circumstances” category) and the cost-control actions (Table 4).

The items in the category of “patients’ circumstances” come from doctors’ observations. Unfortunately, some of the items negatively affect patients’ rights and interests. Our analysis found that some negative circumstances are correlated with the cost-control actions, such as limiting the cost of treating single kind of disease and limiting the conditions for the usage of examinations/drugs/surgery. Obviously, some of the actions described in Table 4, such as limiting drug prescriptions and accepting fewer critically ill patients directly impair patients’ interests by reducing the medical resources that are available for patients, which has consequently lead to reduced patient satisfaction.

**Fig. 1:**
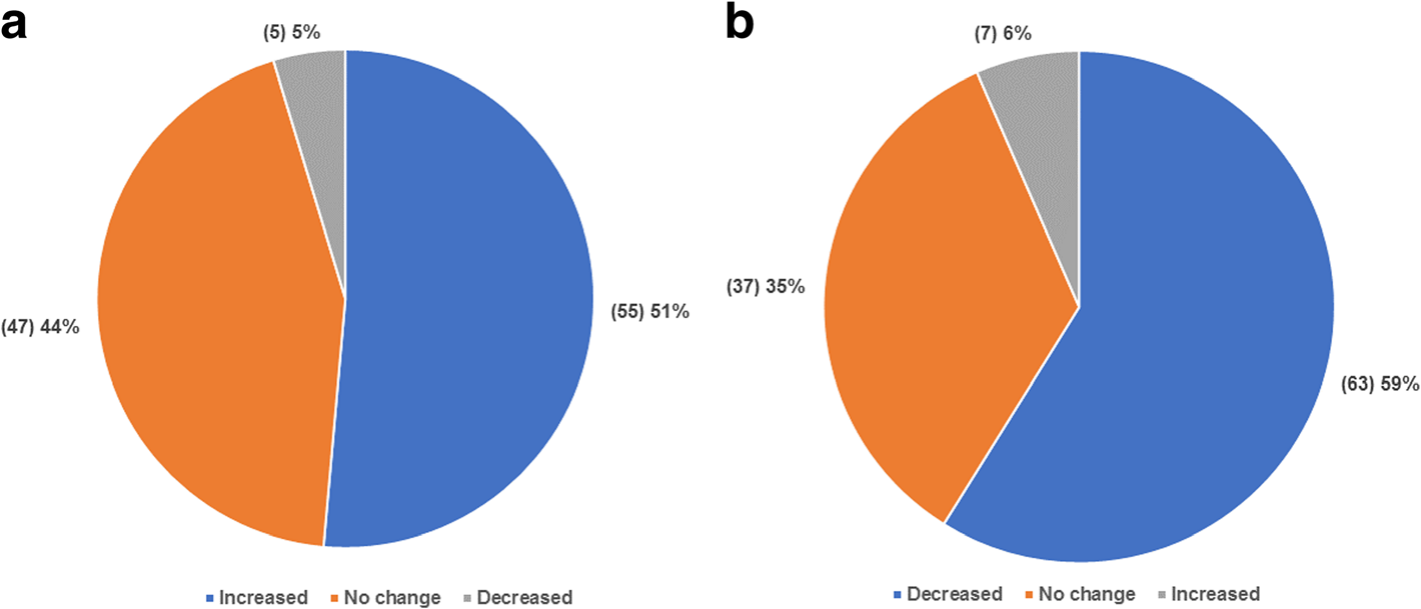
Doctors’ opinions on the changes in the acceptance of patients by hospitals under cost-control actions. **a** Changes in the number of accepted patients without medical insurance and non-local patients accepted by hospitals. **b** Changes in the number of critically ill patients accepted by hospitals

Third, we analysed the relation between patients’ healthcare cost and hospitals’ cost-control actions. Though most of the cost-control actions implemented by hospitals do not directly cause patients to pay more, some of the cost-control actions indirectly enhance patients’ financial burden. For instance, accepting fewer critically ill patients forces those patients to visit other hospitals, which causes those patients to pay more for transportation. This phenomenon is supported by our survey result. Over half of the doctors agreed that hospitals are accepting more patients without medical insurance and non-local patients from other districts since the implementation of the cost-control policy and actions (Fig. 1a). Simultaneously, several doctors also agreed that hospitals are accepting less critically ill patients due to the cost-control actions. Doctors agreed that such circumstances increase patients’ financial burden (Fig. 1b).

Moreover, some of the other cost-control actions increase patients’ financial burden. For example, shortening the duration for prescribed medication and limiting the usage of certain kinds of drugs lead to the consumption of drugs and medications that are not covered by medical insurances. Limiting average prescription costs in outpatient services, limiting the conditions for the usage of examinations/drugs/surgery, and limiting the cost of treating single kind of disease increases the frequency with which patients visit hospitals. Such actions force patients with chronic diseases who require long-term therapies to visit the hospital frequently. The survey showed that rather than helping patients save money, the cost-control actions implemented by hospitals have caused patients to suffer heavier financial burdens. The more ironic fact is that patients are now paying more for worse healthcare services.

Fourth, we analysed the doctors’ answers about the changes in relationship between doctors and patients brought by implementation of the cost-control policy and actions. As described, many of the doctors consider most of the cost-control actions as irrational and restrictive of their abilities to provide effective healthcare services. According to our data, 80% of doctors surveyed are against the policy and actions. For example, hospitals’ actions of limiting the average cost of hospitalization, regularly ranking and limiting the use of top-ranked drugs, and limiting the conditions for the usage of examinations/drugs/surgery are considered irrational not only because they limit doctors’ healthcare performance, but also because they worsen the relation between doctors and patients due to the low quality of healthcare services provided.

Our statistical results found that low patient satisfaction (items in Table 2’s “relationship between doctors and patients” category) is associated with many of the cost-control actions. For example, limiting the conditions for the usage of examinations/drugs/surgery, accepting fewer critically ill patients, and reducing the use of brand-name drugs increases the healthcare costs paid by patients themselves. Some of the actions directly limit patients’ healthcare options and thus lower patient satisfaction (Table 5). Lastly, according to the analysis, the heavier workloads of medical staff, the limited medical resources for patients, and the heavier financial burdens on patients contribute to the worsening of the relationship between doctors and patients. Thus, the analysis suggests that the cost-control policy and actions are negatively affecting both doctors and patients, which are the core components of healthcare systems.

### Multivariate analysis of the network model

**Fig. 2:**
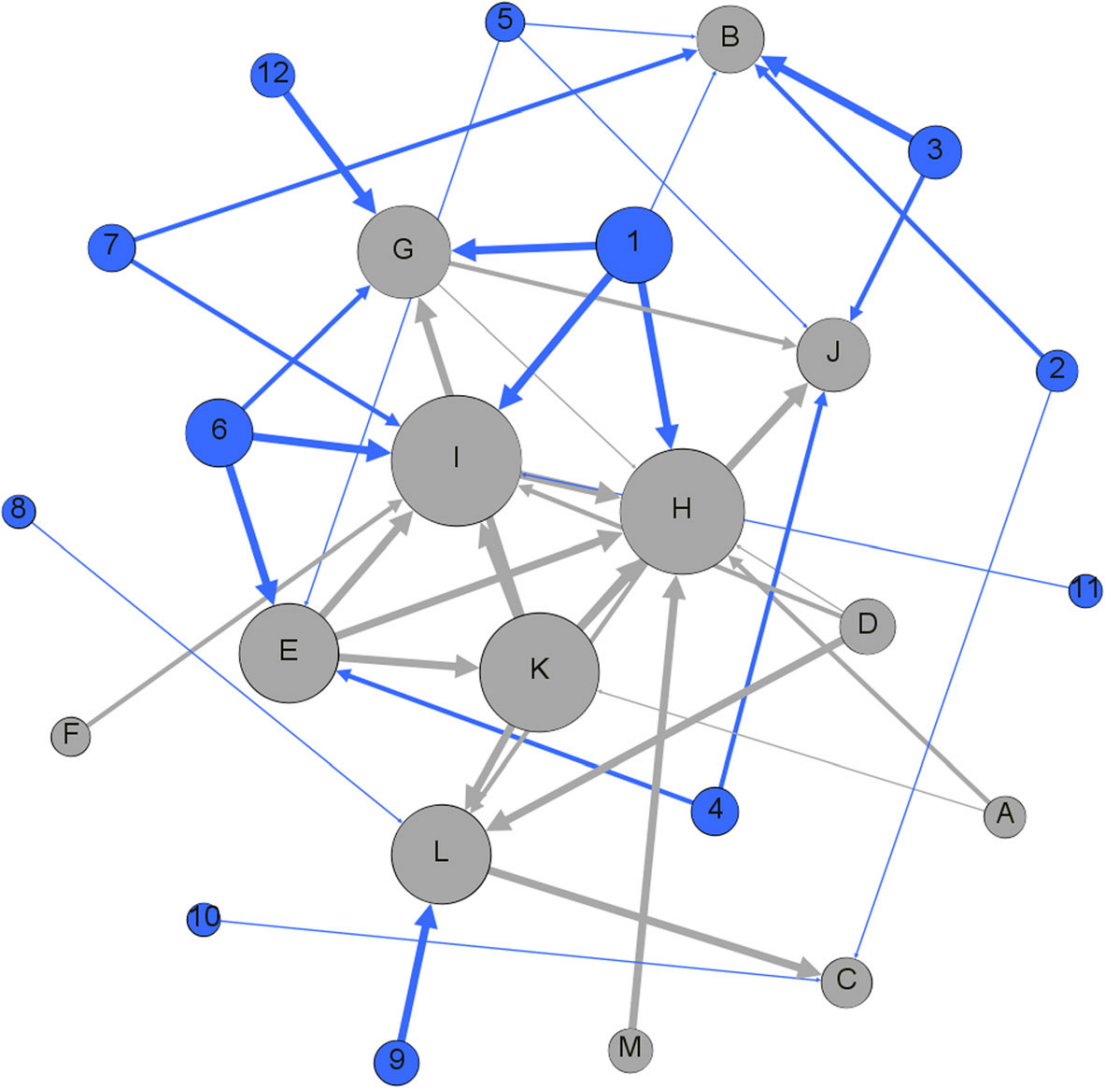
The network model linking between the cost-control actions of hospitals and resultant consequences. Blue nodes are cost-control actions, and the grey nodes are doctors’ opinions. Node labels’ interpretation: 1. Hospitals accept fewer critically ill patients; 2. Limit average cost in hospitalization; 3. Limit average prescription cost in outpatient service; 4. Limit cost of treating single kind of disease; 5. Limit costs and amounts of examinations/drugs /surgery prescriptions; 6. Limit the conditions for the usage of examinations/drugs/surgery; 7. Limit the cost of examination; 8. Limit the duration of hospitalization; 9. Limit the proportional cost of total medical expenses (e.g., the proportion of drug costs); 10. Regularly rank and limit the use of top-ranked drugs; 11. Reduce the use of brand-name drugs; 12. Shortened duration of prescribed medication; A. Higher frequency of visiting hospitals by patients; B. Hospitals’ cost-control actions affect doctors’ healthcare performance; C. Hospitals’ cost-control actions are irrational; D. Hospitals’ cost-control actions increase staff workloads; E. Hospitals’ cost-control actions seriously limit doctors’ healthcare performance; F. Increase the doctors’ explanation and other workloads to patients; G. Indirect costs of patients for visiting hospitals increase (time, transport fees, etc.); H. Less medical resources for patients; I. Lower patient satisfaction; J. The average cost of healthcare paid by patients increases; K. The total cost of healthcare paid by patients increases; L. Worsen the relationship between doctors and patients; M. The limits on drug prescriptions affect the doctors’ healthcare performance

We integrated the results of the bivariate analysis and performed the multivariate analysis on the network model constructed. The data sources of the network model come from the answers of questionnaire and results of the statistical tests, as indicated in the method section. In the network model, components could be connected through diverse types of associations; hence, through topological or other types of network analysis, the key and core component in the entire network system can be found. In this work, we employed such an approach to determine how the cost-control policy and actions can be improved. Based on the results of our analysis, we constructed a network model to visualize the association events of the cost-control policy and actions (Fig. 2). In total, there are 25 nodes and 43 edges in the network. As nodes with greater degrees are set to be bigger in node size, we found that the nodes I, H, K, E, L, and G are with the higher degree in the network, with degree numbers of 9, 9, 7, 6, 6, and 6, respectively. These nodes are a series of the consequences (in grey colour) of the cost-control policy and actions. These consequences include the reduction in medical resources available for patients, reduced patient satisfaction, the increase in total healthcare costs paid by patients, limitations on doctors’ healthcare performance, and the increase in the indirect costs of patients for visiting hospitals (time, transport fees, etc). Poor healthcare quality seriously threatens patients’ health. In fact, the 25 nodes in the network are not independent to each other but they have association to each other as they are the consequences of the implementation of the cost-control policy. Moreover, the results of the multivariate analysis of these associated consequences are consistent and no contradiction points were found, which indicates the validity of the results.

## Discussion

Many of the cost-control actions implemented by hospitals are negatively impacting patients, which is inconsistent with the purpose of public hospitals. Public hospitals ought to provide sound healthcare to patients, but obviously, many of the cost-control actions that hospitals are implementing are lowering the quality of healthcare; hence, patients’ interests are not considered high priority. It is of great surprise that reducing the use of brand-name drugs is taken as a cost-control action in China. In developed countries where the healthcare systems are more mature and better than China’s, brand-name drugs would not be easily replaced by generic drugs because of the differences in therapeutic effects [23–25]. Hence, we feel surprised to find that some of the hospitals in China are replacing brand-name drugs for the purpose of cost saving.

We thought that adopting and implementing global budget for controlling the healthcare costs are more than just cutting the cost down. The quality of healthcare is the much more critical thing to be concerned prior to the cost-saving. Based on foreign studies about global budget and cost-control, and the realistic situations of China, we tried to make recommendations on how China could better implement the global budget for healthcare cost-control. First, China should make flexible payment methods for healthcare costs. Reform of the payment methods plays important role in controlling the increase of the healthcare costs. We consider that, hospitals in China should try to adopt different types of healthcare cost payment. E.g., paying by bed/time usage, paying by disease types, etc. Second, hospitals could motivate the healthcare staffs with incentives. According to Swayne’s study [26], the global budget for cost-control could lower the motivation of the staffs to certain degree, and hence re-motivating staffs by the extra incentive may be a good idea. E.g., hospitals could give extra bonus to staffs who completed the cost-control actions. Third, it is necessary to establish the quality control system of healthcare together with the implementation of global budget cost-control. Given the insufficient monitoring of healthcare practice in China, it is possible that patient would receive less necessary medical resources on implementation of the cost-control actions. Therefore, it would be better if a quality control system could be set up for monitoring the healthcare quality. Fourth, it is important to make clinicians follow the medication guides during healthcare practice. According to the report by Ford et al., following the clinical pathways and medication guidelines could save up to 70% medical resources and improve healthcare quality by up to 50% [27]. Although China has established the basic clinical pathways for parts of the diseases [28], so far clinicians have not been informed to follow the pathways in their practice. Given the great advantages of the clinical pathways in terms of cost-performance, we suggest governmental agencies and hospitals to promote the clinical pathways and guidelines for healthcare practice, and then make clinicians learn and follow the guidelines, since this could achieve the purpose of healthcare cost-control as well. Fifth, the transparent management of the medical insurance funds would be highly helpful. This approach could lead to better communications between fund management institutions and produce efficiency in financial management, so as to contribute to the sustainability of medical insurance funds as well as the healthcare system.

This study can be improved in several ways. First, we only displayed the relevant research outcomes from the perspective of clinical doctors, which does not reflect the perspectives of other groups. In future research, we also plan to investigate the perspective of patients, whose feelings and opinions are equally important to those of the clinical doctors’. Based on doctors’ observation on patients, we also indirectly identified patients’ responses for the cost-control policy and action. Nevertheless, a direct survey on patients in the further study would be an important complement to understand the effects of the cost-control policy and actions. In addition, we could improve the design of the questionnaire with more robust tests of validity and reliability in the future works.

The large population of China has made its healthcare system highly complex. China’s healthcare system is being reformed, and the budget control is a key component in the reform. However, changing and reforming of China’s healthcare system is a tough and complicated long-term project; hence, errors are unavoidable. Therefore, to improve the healthcare system in China, it is necessary to identify and rectify errors while implementing long-term reform.

## Conclusions

We surveyed clinical doctors about the cost-control policy and actions implemented by the hospitals, and we discovered that the actions are considered irrational and negative by many doctors because the actions not only limit the healthcare performance of doctors, but also reduce patients’ medical resources, lower healthcare quality, increase the financial burden of patients, and worsen the relationship between doctors and patients. We performed chi-squared tests and determined the factors relevant to doctors’ opinions and feedbacks. Though our survey samples only covered the key hospitals in several cities of China, we believe that the results of our study are solid and representative, and other hospitals not covered in our survey have the similar issues and circumstances brought by the cost-control policy and actions, too. In summary, the cost-control policy and actions are still far from perfect and there are the spaces for improvement.

## Additional file

Additional file 1: S1 An English Version of the Questionnaire. The survey was conducted in Chinese and the original questionnaire was written in Chinese as well. The English version of sample questionnaire may be slightly different to the original Chinese one due to in-house data processing and translation. (DOCX 32 kb)
