# Supplementary material for: China’s new policy for healthcare cost-control based on global budget: a survey of 110 clinicians in hospitals
Source: BMC Health Serv Res. 2019 Feb 1;19:84. doi: 10.1186/s12913-019-3921-8 (PMC6357408; doi:10.1186/s12913-019-3921-8)
Supplement: Supplementary file 1 — S1 An English Version of the Questionnaire. The survey was conducted in Chinese and the original questionnaire was written in Chinese as well. The English version of sample questionnaire may be slightly different to the original Chinese one due to in-house data processing and translation. (DOCX 32 kb) [file 12913_2019_3921_MOESM1_ESM.docx]

**Questionnaire for Global Budget System Study and Hospital Cost-Control Survey**

**Part I Basic information of interviewee**

Please mark “ X ” in the ○ of your answer.

- Type of your hospital:

○ General hospital; ○ Specialist Hospital;

- Class of your hospital:

○ First-class Grade A;

○ First-class Grade B;

○ Middle-class;

○ Community clinics;

- Type of your professional title:

○ Chief doctor;

○ Associate chief doctor;

○ Attending doctor;

○ Resident doctor;

Inquiry contact:

Jianzhou Yan ([cpuqqyan@163.com](mailto:cpuqqyan@163.com));

Research Center of National Drug Policy and Ecosystem,

China Pharmaceutical University.

**Part II Survey questions**

Please mark “ X ” in the □ or ○ in front of your answer.

**1. Under the global budget system, what kind of cost-control actions below, are taken by your hospital? (Multiple choices in □). And do these cost-control actions affect your healthcare performance?**

□ Limit average prescription cost in outpatient service:

○ Yes, my performance is affected by it; ○ No, my performance is not affected.

□ Limit average cost in hospitalization:

○ Yes, my performance is affected by it; ○ No, my performance is not affected.

□ Limit the duration of hospitalization:

○ Yes, my performance is affected by it; ○ No, my performance is not affected.

□ Limit costs and amounts of examinations:

○ Yes, my performance is affected by it; ○ No, my performance is not affected.

□ Limit cost of treating single kind of disease:

○ Yes, my performance is affected by it; ○ No, my performance is not affected.

□ Regularly rank and limit the use of top-ranked drugs:

○ Yes, my performance is affected by it; ○ No, my performance is not affected.

□ Limit costs and amounts of examinations / drugs / surgery prescriptions:

○ Yes, my performance is affected by it; ○ No, my performance is not affected.

□ Limit the conditions for the usage of examination / drug / surgery:

○ Yes, my performance is affected by it; ○ No, my performance is not affected.

□ Limit the proportional cost of total medical expenses (e.g., the proportion of drug costs):

○ Yes, my performance is affected by it; ○ No, my performance is not affected.

**2. After implementation of your hospital’s cost-control actions, do below changes in healthcare performance happen or not?**

- Duration of prescribed medication:

○ is shortened; ○ is not shortened.

- Usage of brand-name drugs:

○ is reduced; ○ is not reduced.

- Healthcare workload:

○ is increased, e.g., more explanations to patients (about changes in healthcare decisions).

○ is not increased.

**3. After implementation of your hospital’s cost-control actions, do below changes happen to patients or not, according to your observation?**

- Frequency of Patients’ visit to hospital:

○ is higher; ○ is not higher.

- Medical resources for patients:

○ become less; ○ do not become less.

- Average cost of healthcare paid by patients:

○ is increased; ○ is not increased.

- Total cost of healthcare paid by patients increases:

○ is increased; ○ is not increased.

- Indirect costs of patients for visiting hospitals (time, transport fees, etc.):

○ is increased; ○ is not increased.

**4. After implementation of your hospital’s cost-control actions, according to your opinion, please answer below questions regarding to relationship between doctors and patients, as well as the patient acceptance condition.**

- Satisfaction degree of patients:

○ is lower; ○ is not lower.

- Relationship between doctors and patients:

○ becomes worse; ○ does not become worse

- Acceptance of critically ill patients by your hospital:

○ is affected by hospital’s cost-control actions;

○ is not affected by hospital’s cost-control actions.

- The number of critically ill patients accepted by your hospital:

○ is decreased; ○ is not decreased.

- Acceptance of patients without medical insurance or non-local patients:

○ is affected by hospital’s cost-control actions;

○ is not affected by hospital’s cost-control actions.

- The number of patients without medical insurance or non-local patients accepted by your hospital:

○ is decreased; ○ is not decreased.

**5. Please answer questions below based on your comprehensive observation and feeling to your hospital’s cost-control actions:**

- Are your hospital’s cost-control actions rational?

○ Yes, they are rational; ○ No, they are not rational.

- Do your hospital’s cost-control actions affect doctors’ healthcare performance?

○ Yes, they do; ○ No, they do not.

- Do your hospital’s cost-control actions seriously limit doctors’ healthcare performance?

○ Yes, they do; ○ No, they do not.

- Do your hospitals’ cost-control actions increase workloads of staffs (including doctors’ and other types of healthcare staffs)?

○ Yes, they do; ○ No, they do not.

Note: the survey was conducted in Chinese and the original questionnaire was written in Chinese as well. This English version of sample questionnaire was translated from the original one in Chinese.
